# Structural and functional substrates of tetanus toxin in an animal model of temporal lobe epilepsy

**DOI:** 10.1007/s00429-013-0697-1

**Published:** 2014-01-18

**Authors:** Alex S. Ferecskó, Premysl Jiruska, Lucy Foss, Andrew D. Powell, Wei-Chih Chang, Attila Sik, John G. R. Jefferys

**Affiliations:** 1 Neuronal Networks Group, School of Clinical and Experimental Medicine, College of Medical and Dental Sciences, University of Birmingham, Birmingham, B15 2TT UK; 2 Department of Developmental Epileptology, Institute of Physiology, Academy of Sciences of Czech Republic, 14220 Prague, Czech Republic; 3 Department of Neurology, Charles University, 2nd School of Medicine, University Hospital Motol, 15006 Prague, Czech Republic; 4 Present Address: UCB Pharma, 216 Bath Road, Slough, Berkshire, SL1 3WE UK

**Keywords:** Epilepsy, Synaptic function, VAMP, Tetanus neurotoxin, Hippocampus

## Abstract

The effects of tetanus toxin (TeNT) both in the spinal cord, in clinical tetanus, and in the brain, in experimental focal epilepsy, suggest disruption of inhibitory synapses. TeNT is a zinc protease with selectivity for Vesicle Associated Membrane Protein (VAMP; previously synaptobrevin), with a reported selectivity for VAMP2 in rats. We found spatially heterogeneous expression of VAMP1 and VAMP2 in the hippocampus. Inhibitory terminals in stratum pyramidale expressed significantly more VAMP1 than VAMP2, while glutamatergic terminals in stratum radiatum expressed significantly more VAMP2 than VAMP1. Intrahippocampal injection of TeNT at doses that induce epileptic foci cleaved *both* isoforms in tissue around the injection site. The cleavage was modest at 2 days after injection and more substantial and extensive at 8 and 16 days. Whole-cell recordings from CA1 pyramidal cells close to the injection site, made 8–16 days after injection, showed that TeNT decreases spontaneous EPSC frequency to 38 % of control and VAMP2 immunoreactive axon terminals to 37 %. In contrast, TeNT almost completely abolished both spontaneous and evoked IPSCs while decreasing VAMP1 axon terminals to 45 %. We conclude that due to the functional selectivity of the toxin to the relative sparing of excitatory synaptic transmission shifts the network to pathogenically excitable state causing epilepsy.

**Electronic supplementary material:**

The online version of this article (doi:10.1007/s00429-013-0697-1) contains supplementary material, which is available to authorized users.

## Introduction

Tetanus neurotoxin (TeNT) is a remarkably potent *Clostridial* toxin which in the spinal cord causes spastic paralysis (Pellizzari et al. 1999), and in the forebrain epileptic seizures (Yeh et al. 2010; Jefferys and Walker 2006). Its light chain is a zinc protease selective for Vesicle Associated Membrane Protein (VAMP; synaptobrevin), while the heavy chain is necessary for uptake into neurons (Schiavo et al. 2000). VAMP is essential for synaptic transmission so that its proteolysis by TeNT, or by four of the seven botulinum neurotoxins (BoNT/B, D, F and G), causes loss of neurotransmitter release (Schiavo et al. 1992; Dolly et al. 2009). The remaining BoNTs target different synaptic proteins, SNAP-25 and syntaxin, but also block transmitter release. The clinical consequences of intact TeNT (i.e. heavy and light chains linked by disulphide bonds) differ from those of intact BoNT, which causes flaccid paralysis in the spinal cord and BoNT/E has been shown to have antiepileptic actions in the brain (Bozzi et al. 2006). These observations suggest that extracellular intact TeNT has specificity for inhibitory neurotransmission, as shown specifically in “synaptic bouton preparations” of isolated spinal neurons (Shin et al. 2012), while BoNT causes a less specific block of synaptic transmission.

Intrahippocampal TeNT provides a chronic model of temporal lobe epilepsy (TLE) characterised by intermittent spontaneous seizures starting after a latent period of ~7 days (Jiruska et al. 2010, 2013; Jefferys 1989). The diversity of clinical epilepsies argues for diverse experimental models to investigate mechanisms and to develop novel treatments. TLE is a common epilepsy that can be difficult to treat pharmacologically. TLE can be subdivided in various ways, for instance the presence and extent of lesions such as hippocampal sclerosis. For most purposes spontaneous seizures are an important feature of experimental models (Stables et al. 2003). The TeNT model differs from other spontaneously seizing rat TLE models in lacking initial status epilepticus (prolonged epileptic seizures) and produces minimal or no neuronal death in 70–90 % cases with highly focal lesions in the remainder (Jiruska et al. 2010). We previously found that the inhibitory neurotransmitter gamma aminobutyric acid (GABA) release is disrupted within a few days, fully recovering by 8 weeks (Jefferys et al. 1991; Empson and Jefferys 1993; Whittington and Jefferys 1994). The decrease in GABA release during the active seizure phase was selective for the Ca^2+^-dependent fraction (Jefferys et al. 1991). The lack of changes in neuronal responses to exogenous GABA argues for preservation of GABA_A_ receptors (Whittington and Jefferys 1994). More subtle inhibitory dysfunction persisting at least 5 months (Vreugdenhil et al. 2002).

VAMP exists in multiple isoforms, VAMP1 and VAMP2 occur widely in the brain and are involved in synaptic transmission. VAMP2 is cleaved by TeNT (Chen et al. 2012; Mainardi et al. 2012). While VAMP1 is cleaved by BoNT/F (Verderio et al. 1999), it is reported to be resistant to TeNT, at least in the rat (Schiavo et al. 1992, 1993). One hypothesis for the functional specificity of TeNT (Shin et al. 2012) is that it has selectivity for a VAMP isoform primarily expressed in GABAergic synapses. Two alternative hypotheses are that the greater activity of interneurons results in greater uptake of TeNT (Williamson et al. 1992) or that the molecular determinants of presynaptic uptake and intracellular trafficking select for inhibitory synapses (Schiavo et al. 2000). In neocortex VAMP2 occurs in up to twice as many excitatory terminals as VAMP1, while both isoforms are equally represented in inhibitory terminals (Bragina et al. 2010). Previous work on changes in VAMP following intracortical injection of TeNT has only examined VAMP2 and not VAMP1 (Mainardi et al. 2012). Injection of a small amount of TeNT into the hippocampus is extensively used as a model of temporal lobe epilepsy (Jefferys and Walker 2006). Neo- and archicortical areas differ in structure, cell types and functions, so that extrapolation from neocortical data to explain the toxin action in this temporal lobe epilepsy model can be misleading and leaves the possibility open that cell specific expression of VAMP isoforms may allow TeNT to selectively target inhibitory neurons. In the present study, we investigate the selectivity of expression of VAMP isoforms in excitatory and soma-targeting inhibitory synapses in hippocampus, and the impact of intrahippocampal injection of TeNT on the presence of VAMP in the tissue and on synaptic activity in brain slices over a period 2–16 days after injection.

## Methods

### Animals and tetanus toxin injection

Adult male Sprague–Dawley rats weighing 220–280 g were housed under standard conditions with temperature controlled to 22 ± 1 °C and 12/12 h light/dark cycle. The animals had ad libitum access to food and water. All experiments were performed under the Animals (Scientific Procedures) Act 1986 of the United Kingdom and institutional ethical review. Surgery was performed under isoflurane anaesthesia. One microliter of TeNT or vehicle solution (0.05 M phosphate-buffered saline, PBS, Sigma UK, with 2 % bovine serum albumin, BSA, Sigma UK) was injected through a burr hole into the right hippocampus into stratum radiatum of CA3, at coordinates 4.1 mm caudal and 3.9 mm lateral to bregma and 3.8 mm below cortical surface (Paxinos and Watson 1998), at 200 nl/min using a Hamilton microsyringe and infusion pump (KDS311, KD Scientific Inc., USA). The microsyringe was left in place for 5 min to avoid backflow up the injection track. Differences in potency of our stocks meant we injected 25 ng of TeNT for the main immunohistochemical studies (Sigma-Aldrich #B5002, Gillingham, UK) and 2.5–5.0 ng for electrophysiology and its associated immunohistochemistry (#190A, Quadratech Diagnostic Ltd, Epsom, UK). Analgesic (Buprenorphine, 1 ml/kg) was injected intramuscularly during surgery. Animals were housed individually.

Some rats were used to determine where seizures were initiated with respect to the injection site. Following the injection, a square craniotomy was made centered at −4.2 mm anterio-posteriorly and 3.1 laterally from bregma. Twelve movable tetrodes were implanted through the craniotomy into the neocortex above the dorsal hippocampus (Fig. 7). Tetrodes were fabricated from tungsten wire 12 μm in diameter (H-Formvar insulation with Butyral bond coat, California Fine Wire, Grover Beach, USA). The tips of the electrodes were gold plated to reduce the electrode impedance to <300 kΩ. Following the implantation, craniotomy was sealed with paraffin. Reference and ground electrodes were inserted into the bone above the cerebellum. A microdrive assembly was then embedded in dental cement and attached to the skull with anchoring screws. After 1 week to allow recovery, electrodes were slowly lowered into the hippocampus. Spontaneous brain activity was recorded daily starting on day 8 and continued for 4 weeks. Each recording session lasted 2–8 h of continuous recording. Signals were pre-amplified to minimize the movement artifacts, amplified (100× and 500×), band-pass filtered at 0.1–5,000 Hz and digitised at 24 kHz using an Axona recording system (Axona Ltd, St. Albans, UK).

For immunohistochemistry, animals were divided into three survival groups: 2 days (6 TeNT; 3 controls), 8 days (6 TeNT; 2 controls) and 16 days (6 TeNT; 2 controls). None of the experimental measures in the controls differed significantly between the three survival times (ANOVA, *P* > 0.05), so to meet obligations to minimize animal numbers we pooled them for subsequent analyses. Animals surviving 8 and 16 days were video-monitored continuously from the 5th day postinjection using infrared digital cameras (MSI 380i, Taipei City, Taiwan) and Spike 2 software (CED, Cambridge, UK) to verify the development of spontaneous recurrent seizures. At the end of the experiments animals were overdosed by anaesthetic (ketamine–medetomidine) and perfused transcardially with 4 °C saline (0.9 % NaCl, Sigma, UK) followed by 4 % paraformaldehyde in 0.1 M phosphate buffer (PB, pH 7.4, Sigma, UK). Brains were extracted and postfixed 1 h in 4 % paraformaldehyde and then transferred into 0.1 M PB for sectioning.

### Histology and immunohistochemistry

For immunofluorescence labelling, 80-μm-thick coronal sections were made using a vibratome (Model 1000 Pelco Int., Redding, CA, USA). The sections were rinsed in 0.1 M PB (pH 7.4) 2 × 15 min to remove unbound fixative and washed in Tris buffer saline (TBS, 0.05 M, Sigma, UK) 3 × 20 min containing 0.05 % Triton X-100 (pH 7.6). Incubation of the sections in primary and secondary antibodies was performed under continuous, gentle agitation at 4 °C. For double immunolabelling pairs of commercially available antibodies, polyclonal anti-VAMP1 (rabbit; 1:200, Abcam, Cambridge, UK), or anti-VAMP2 (rabbit, 1:1,000, Synaptic Systems, Goettingen, Germany) and anti-GAD 65 (GAD-6) (mouse, 1:3,000 Abcam, Cambridge, UK) or anti-VGLUT1 (guinea pig, 1:1,000, Chemicon, Billerica, USA) and monoclonal anti-Neurofilament 68 clone NR4 (mouse, 1:400, Sigma, UK) were used. Antibodies have been fully characterised and extensively used previously in publications [e.g. VAMP1 (Coller et al. 2012); VAMP2 (Orenbuch et al. 2012)]. Slices were preincubated in a blocking solution containing 5 % of normal goat serum (Vector Laboratories, Peterborough, UK) in TBS and 0.5 % Triton 100-X for 45 min at room temperature to reduce nonspecific background labelling. Sections were then incubated for 3 days at 4 °C in the primary antibodies at the dilutions stated above, in TBS containing 2 % normal goat serum and 0.5 % Triton X-100 and washed with TBS, sections were incubated overnight at 4 °C with secondary antibodies anti-rabbit Alexa Fluor 488 (1:1,000, Molecular Probes, Paisley, UK), CY3 conjugated goat-anti-mouse (1:500, Molecular Probes, Paisley, UK) or goat-anti-guinea pig CY3 (1:500; Jackson Immunoresearch, West Grove, USA). After three washes with PB, sections were mounted on glass slides, air dried at room temperature and coverslipped with homemade anti-fading mounting media (Mowiol).

### Imaging and analyses

Immunofluorescent labelling was investigated using 40× objective and high-resolution 16-bit grey scale images were captured for signal intensity analyses using an Olympus BX61 microscope equipped with an EXi Blue (QImaging, Surrey, Canada) digital camera. The quantification of the immunofluorescent labelling and colocalization analyses were performed with the aid of image acquisition and analyses software ImagePro Plus 7.0 and the AutoQuant X, image deconvolution and 3D Visualization software (Media Cybernetics Inc, Bethesda, MD, USA). To avoid signal fading and therefore altering signal intensity of the immunofluorescent labelling in the final preparations, care was taken to complete micro photographing in one session and to avoid exposing the sections for prolonged periods to UV light or to repeat the capturing process on the same slide.

### Quantitative evaluation of immunofluorescent labelling

To estimate the intensity of immunolabelling and provide comparable dataset for quantitative analyses, constant exposure parameters (illumination strength, exposure time, etc.) were preserved throughout the entire process within and between animal groups. VAMP immunolabelling in each slice was normalized by the labelling intensity in contralateral subiculum. This region was selected for normalization because: (1) it consistently showed high labelling intensity; and (2) it lacks direct anatomical connections with the injected hippocampus and should not be affected by the toxin application. Normalized immunolabelling was plotted distributed into eight bins of intensity (grey scale). We analysed individual hippocampal subregions (CA1, CA3, DG) of both hemispheres. The number of pixels in each grey scale bin was plotted as a percentage of the total surface (i.e. all bins) of the analysed region. This process generated intensity spectrum graphs for each region of each slice (Supplemental Figure 1). The first moment *mom*_1_ of the grey scale spectrum is a weighted average of the intensity of staining and was calculated using the formula

$$mom_{1} = \frac{{\sum {f \cdot I} }}{\sum I }$$

where *f* is grey colour bin from grey colour spectrum and *I* is its proportion it occupies from the total area.

### Quantification of VAMP immunopositive boutons

The grey scale analysis of VAMP labelling has two limitations: it is unlikely to be linearly related to levels of VAMP in the tissue and it requires relatively large areas for analysis. Therefore, we also quantified VAMP immunoreactive boutons. Z-stack images spanning the 2 μm from the surface were acquired, underwent 3D deconvolution using AutoQuant (MediaCybernetics, MD, USA) to reduce the distortion and produce a clearer image, and then flattened using Extended Depth of Field for maximum intensity to obtain a single image (ImagePro Plus, MediaCybernetics, MD, USA). Filters were applied to enhance the image and aid the automatic counting of the boutons. A region of interest (sized 100 μm^2^) was placed in four different areas across the image, and the software automatically counted the bright objects in the area followed by a watershed split giving an output of all the different areas of the counted boutons. Only those objects that were within the size range of 0.1–0.7 μm^2^ were included in the analysis as boutons. The number of boutons was measured in four regions of interest for each image and averaged.

### Colocalization analysis

Z-stack images covering 2 μm from the surface were acquired, underwent 3D deconvolution using AutoQuant (MediaCybernetics, MD, USA). To measure colocalization between immunosignals at the single bouton level line histograms were created in randomly chosen areas in or adjacent to the pyramidal cell layer. Quantitative colocalization measurements used for statistical analysis were performed on each pixel in the area of interest. To prevent false positive and negative results due to photobleaching (slices at the bottom of the stack) and overexposure (slices at the top of the stack) multiple (>15) optical stacks were taken from the top 2 μm and an average of five individual optical slices from each Z stack of images were used for quantitative colocalization analysis. Measurements were performed only once on a given area. ImagePro’s built-in colocalization algorithm was used to measure Pearson’s coefficient to evaluate colocalization at the population level, and line measurement to analyse colocalization at the bouton level (ImagePro Plus, MediaCybernetics, MD, USA). The quantitative colocalization analysis was performed on the entire optical section without defining any area of interest.

### Electrophysiology

Electrophysiology was performed 8–16 days following TeNT injection, when the changes in VAMP immunohistochemistry were greatest. Animals were anaesthetized by ketamine/medetomidine and transcardially perfused with sucrose solution. Brains were removed and 300-μm coronal hippocampal slices were prepared and placed in a holding chamber. For recording, slices were transferred to a submerged chamber. Whole-cell patch-clamp recordings were made from the somata of CA1 pyramidal neurons from the CA2 and subicular ends of the CA1 region of ipsilateral and contralateral hippocampi. Electrophysiological recording started 10 min after establishment of the whole-cell configuration. Pyramidal neurons were identified using an infrared differential interference contrast on an Olympus BX-51 upright microscope, fitted with a fluorplan 40×, 0.8 NA water immersion lens (Micro Instruments, Long Hanborough, UK). CA1 neurons were visually selected based on appearance and the integrity of the apical dendrites (visible for ~100 μm from the soma). Patch electrodes were pulled from borosilicate glass (OD 1.2 mm, ID 0.69 mm; Harvard Apparatus, Edenbridge, UK) using a P-97 puller (Sutter, Novato, CA, USA). The intracellular solution comprised (mM); 135 CsCH_3_SO_4_, 8 NaCl, 10 HEPES, 5 QX-314, 2 Mg-ATP and 0.3 Na-GTP; pH 7.3 with KOH (osmolarity ~285 mOsm); 0.5 % neurobiotin enabled identification of the recorded cell and correlation of its synaptic activity with loss of VAMP1 and 2. EPSCs were recorded at −75 mV to avoid contamination by IPSCs, and were confirmed as EPSCs by their abolition by 20 μM NBQX and 25 μM d-APV (not shown). IPSCs were recorded at 0 mV to avoid contamination by EPSCs, and were confirmed as IPSCs by their abolition by 10 μM bicuculline (not shown). Patch electrodes typically had resistances of 4–7 MΩ. Membrane potentials and currents were recorded using an NPI SEC-10L amplifier (Scientifica, Harpenden, UK) or Axoclamp 2 (Molecular Devices, Sunnyvale, CA, USA), low-pass Bessel filtered at 1 kHz (NL-125, Digitimer Ltd, Welwyn Garden City, UK) and digitised at 10 kHz by a Power 1401 (CED Ltd, Cambridge, UK). Stimulation and data acquisition were controlled using Signal software (version 3; CED). Evoked postsynaptic currents were generated using a glass stimulating electrode placed in stratum radiatum (~50 μm from stratum pyramidale and 50 μm from the recorded cell). Spontaneous synaptic events were analysed using MiniAnalysis (version 6.0.3, Synaptosoft, Decatur, GA, USA). The threshold for synaptic event detection was set at 5× root mean baseline square noise, which averaged 2.97 ± 0.20 pA.

### Immunohistochemical analysis of in vitro slices

After recording, slices were incubated in 4 % PFA for 1.5 h, then washed in 0.1 M PB and stored in 0.1 M PB with thimerosal (1:1,000) at ~4 °C before sectioning. Each 300-μm slice was resectioned to 80 μm using a Vibratome (Model 1000 Pelco Int., Redding, CA, USA) and stored in 0.1 M PB and thimerosal (1:1,000) before immunohistochemistry (VAMP1, VAMP2, neurobiotin). Sections to be incubated with the VAMP1 antibody underwent a further 5 h incubation in 4 % paraformaldehyde to achieve optimal staining. The staining procedure was carried out in the same way as above, with the addition of streptavidin conjugated Alexa 546 (1:200) to the secondary antibody solution, to visualise neurobiotin in recorded cells. After immunolabelling for VAMP and neurobiotin, low magnification images were taken of the VAMP1/2 labelling and the position of the neurobiotin-containing cell. This was followed by high magnification images using an oil immersion lens around the portion of the stratum radiatum directly adjacent to stratum pyramidale and the labelled cell.

### Statistical analyses

The statistical analyses specified in the text were applied using SPSS software, with a significance criterion of 0.05. Results are expressed as a mean and standard error of the mean (SEM) unless otherwise stated.

## Results

### Behavioural effect of tetanus toxin injection

It is already well established that injecting TeNT into rat hippocampus induces epilepsy with a latency of approximately 7 days following the injection. Seizure semiology in the present study corresponded to complex partial seizures of limbic origin with secondary generalization as described previously (Finnerty and Jefferys 2000): episodes of prolonged staring, orofacial automatisms followed by forelimb clonus, rearing, falling and generalised tonic–clonic movements and frequently followed by wet dog shakes.

### Distribution of the VAMP1 and VAMP2 protein in the rat hippocampus

**Fig. 1:**
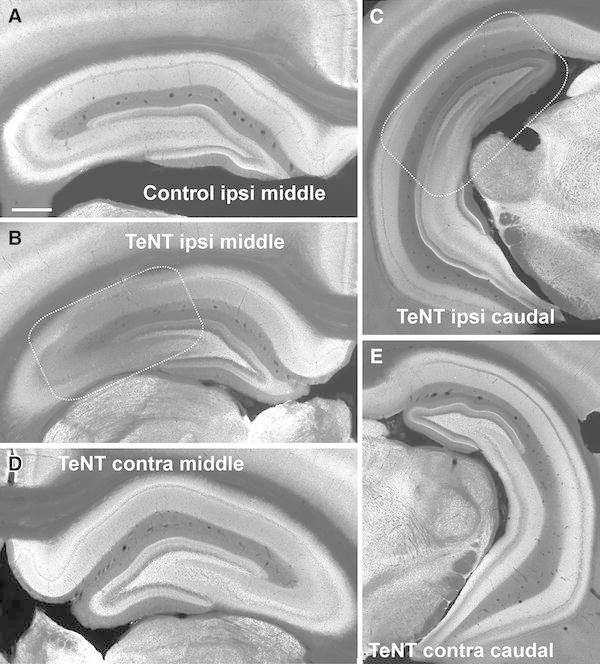
VAMP1 immunofluorescent labelling in control and TeNT-treated hippocampus. **a** Distribution of VAMP1 in normal dorsal hippocampus. **b** Depletion of VAMP1 in the middle parts of the hippocampus 8 days after the tetanus toxin (TeNT) injection.* Dashed lines* mark the approximate area of decrease in VAMP1 labelling. **c** On day eight the VAMP1 deficit is observable also in caudal parts of the injected dorsal hippocampus. Middle (**d**) and caudal parts (**e**) of contralateral hippocampus are not affected by VAMP1 deficit. *Scale bar* (**a**) 1 mm

**Fig. 2:**
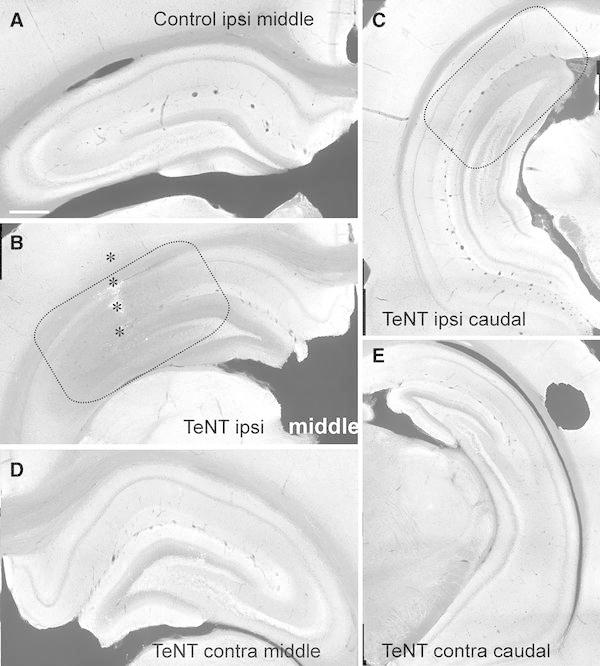
VAMP2 immunofluorescent labelling in control and TeNT-treated hippocampus. **a** Distribution VAMP2 in the dorsal hippocampus of control animals. **b** Deficit in VAMP2 immunolabelling in the middle part of the hippocampus 8 days following the tetanus toxin (TeNT) injection. *Dashed lines* mark the approximate area of decrease in VAMP2 immunolabelling. *Stars* mark the glial scar after the needle track. **c** On day eight the VAMP2 deficit also is observable in caudal parts of the injected hippocampus. Middle (**d**) and caudal parts (**e**) of contralateral hippocampus do not show VAMP2 depletion. *Scale bar* 1 mm

**Fig. 3:**
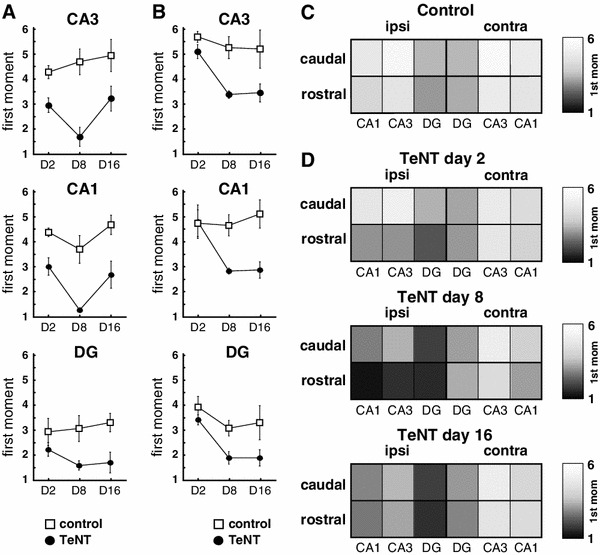
Spatiotemporal changes in VAMP1 immunofluorescence. **a** Temporal changes of the first moment of VAMP1 immunolabelling intensity in individual hippocampal subregions of middle parts of the injected hippocampus. In CA3 and CA1 the shift towards the darker bins is present on day 2 suggesting loss of VAMP1 immunosignal. **b** Temporal profile of changes of the first moment of the labelling intensity in caudal parts of ipsilateral hippocampus. First changes occur on day 8. **c** *Grey scale* map representation of the mean values of the first moments of VAMP1 immunolabelling intensity in individual regions of ipsilateral and contralateral hippocampi of control animals. **d** *Grey scale* plot of mean values of first moments of VAMP1 immunolabelling in TeNT animals during day 2, 8 and 16. It demonstrates early shift towards lower values of the first moments (*darker colours*) in ipsilateral middle parts of the hippocampus and later decrease in VAMP1 immunolabelling in caudal parts of the hippocampus

The two isoforms had distinctive, but overlapping, distributions in the hippocampal formation of control rats. At low magnification VAMP1 showed high labelling intensity (bright signal) in the strata oriens and radiatum of CA1 and CA3 and the inner molecular layer of dentate gyrus (DG). VAMP1 immunolabelling was weak in the hippocampal fissure, in stratum lacunosum moleculare of CA1 and outer molecular layer of DG (darker layers, Fig. 1a). VAMP2 immunolabelling was more evenly distributed than VAMP1 (compare panels A in Figs. 1 and 3). Labelling of VAMP2 was greater in the dendritic layers of CA1, CA3 and DG, especially in CA1 stratum lacunosum molecular and DG outer molecular layer, with almost no variations in labelling intensity throughout the rostro-caudal axis of the hippocampus. VAMP2 also was particularly strong in stratum lucidum of the CA3 indicated by the presence of a discrete band of intensely labelled axon terminals, presumably deriving from mossy fibres adjacent to the CA3 pyramidal layer (Fig. 2a). The intensity of VAMP2 immunolabelling was low in the stratum pyramidale of CA1 and CA3. The weakest labelling of both isoforms was in the granule cell layer of the DG.

### Spatiotemporal profile of tetanus toxin action on VAMP1

Injection of TeNT resulted in decreased VAMP1 labelling (Figs. 1b, c, 3). Two days after TeNT injection, the loss of VAMP1 was modest and highly localized close to the injection site, so that it mostly affected CA3 and adjacent parts of CA1 and DG in the rostral hippocampal section (not shown). The loss of VAMP1 became more substantial and extensive (2.5 ± 0.2 mm, *n* = 5) eight days after injection (Figs. 1b, c, 3) and started to recover by 16 days (Fig. 3). The TeNT-induced decrease in VAMP1 can be assessed by the labelling-intensity spectrum graphs by a shift of the peaks towards darker bins from those for controls. To quantify the spatiotemporal profile we calculated the first moment of the labelling-intensity spectra (Fig. 3). Results showed that loss of VAMP1 appeared by 2 days in rostral CA3 and CA1, and decreased further by day 8 (Fig. 3a). The loss is sustained at day 16, but started to recover in the middle section CA1 and CA3 (Fig. 3a). This spatiotemporal profile is consistent with the first loss of signal appearing close to the injection site and then spreading to the dentate gyrus and to the caudal sections through the hippocampus (Fig. 1b). There is no evidence of changes in VAMP1 immunostaining intensity contralateral to the injection (Fig. 2d, e).

### Spatiotemporal profile of tetanus toxin action on VAMP2

**Fig. 4:**
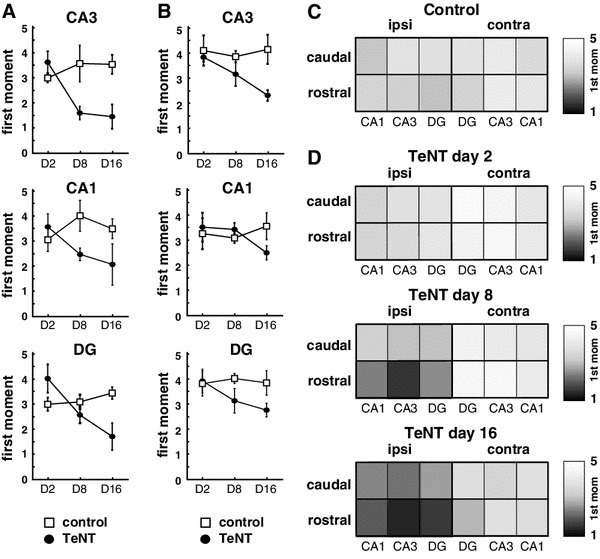
Spatiotemporal changes in VAMP2 immunofluorescence. **a** Temporal changes of the first moment of VAMP2 immunolabelling intensity in individual subregions of the middle dorsal hippocampus. Shift towards the lower mean values of the first moment correspond to decrease in VAMP2 immunolabelling. The decrease is most pronounced on days 8 and 16. **b** Temporal profile of changes of the first moment of VAMP2 immunolabelling intensity in caudal parts of ipsilateral hippocampus. First changes occur on day 16 in CA3. **c** *Grey scale* map representation of the mean values of the first moments of VAMP2 immunolabelling intensity in individual regions of ipsilateral and contralateral hippocampi of control animals. **d** *Grey scale* plot of mean values of first moments of VAMP2 immunolabelling in TeNT animals during day 2, 8 and 16. Shifts towards low values of the first moments (*darker colours*) are present in ipsilateral hippocampus and while contralateral hippocampus is spared

The effects of TeNT on VAMP2 expression 8 days after injection are shown in Fig. 2. By this stage the area of reduced VAMP2 signal covered large parts of ipsilateral CA1 and CA3, and the lateral parts of DG (Fig. 2b, c), and there is some evidence of decreased VAMP2 contralaterally, particularly in caudal CA3 (Fig. 2d). The analysis of spatiotemporal changes in VAMP2 (Fig. 4) shows that the decrease of the immunosignal starts later than 2 days postinjection, first being seen at day 8 in CA1 and CA3 of rostral ipsilateral sections, extending to all regions by day 16.

### Neuronal specific expression of VAMP1 and VAMP2

**Fig. 5:**
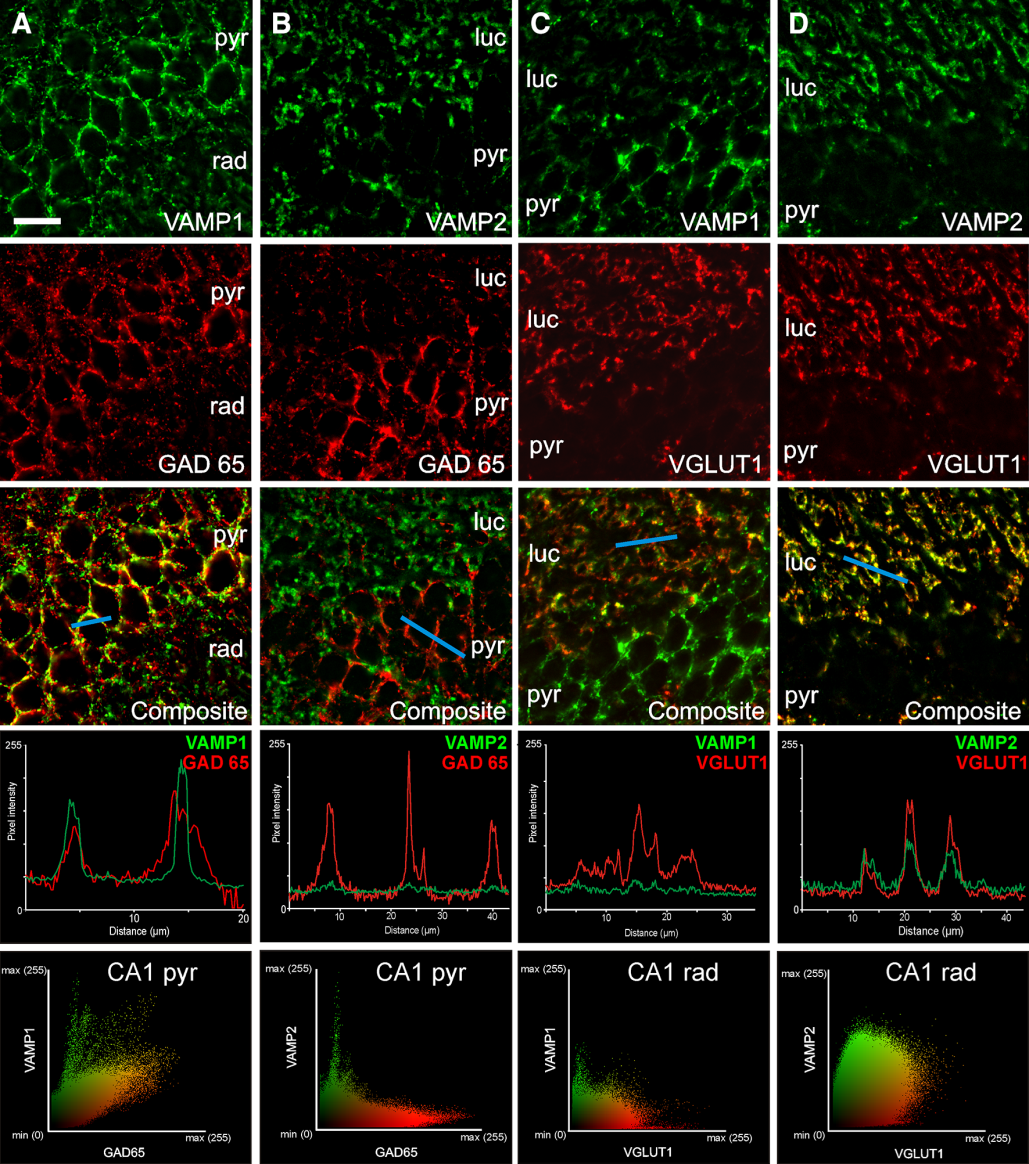
Neuronal specific expression of VAMP1 and VAMP2 assessed by colocalization with putative inhibitory (GAD65) and excitatory (VGLUT1) markers in control animals. **a** Immunofluorescent microphotographs depict strong colocalization between VAMP1 and GAD65 immunolabelled axon terminals. **b** VAMP2 and GAD65 positive axon terminals show large degree of spatial segregation in strata pyramidale and lucidum. **c** VAMP1 and VGLUT1 immunopositive terminals show weak colocalization in the strata lucidum and pyramidale. **d** Immunolabelling for VAMP2 and VGLUT1 terminals demonstrate strong colocalization particularly in stratum lucidum. *pyr* stratum pyramidale, *luc* stratum lucidum, *rad* stratum radiatum. Line profile analysis in the *fourth row* indicates signal overlap between VAMP1-GAD65 and VAMP2–VGLUT1 double immunolabelled axon terminals, and spatial segregation of VAMP1-VGLUT1 and VAMP2-GAD65 immunoreactive boutons. *Blue bars* on the composite microphotographs (*row 3*) show the site and length of line measurement. *Coloured scatter plots* in the *bottom row* represent distribution of co-localizing pixels according to intensity in the CA1 strata pyramidale and radiatum, respectively. *Scale bar* 20 μm

**Fig. 6:**
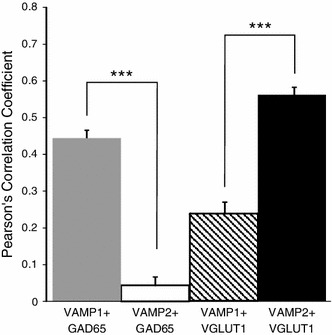
Quantitative analysis of coexpression of VAMP1, VAMP2, GAD65 and VGLUT1 protein in axon terminals. Pearson’s correlation is shown for each VAMP isoforms double labelled with GAD65 and VGLUT1, respectively. Significantly higher overlap was detected of VAMP1–GAD65 labelling compared to VAMP2 isoform (***P < 0.0001, unpaired *t* test, *r* ^2^ = 0.79). Colocalization between VAMP2–VGLUT1 was significantly higher than VAMP1–VGLUT1 (***P < 0.0001, unpaired *t* test, *r* ^2^ = 0.77). Data are given as mean ± SEM

We investigated the neuronal specificity of the expression of the two VAMP isoforms. Excitatory and inhibitory cells and their synapses can be directly characterised by the presence of specific transmitter synthesizing or transporting enzymes, VGLUT1 and GAD65, respectively. To differentiate VAMP1 and VAMP2 expression between excitatory and inhibitory axon terminals we performed double immunolabelling for each VAMP isoform and GAD65 (for inhibitory) and VGLUT1 (for excitatory). Cellular differences in the expression of the two isoforms were found in CA3 (Fig. 5) and CA1. VAMP1 strongly co-localized with GAD65 positive boutons, particularly in the mainly soma-targeting boutons of stratum pyramidale, which play a key role in regulating pyramidal cell firing and hence in epilepsy (Fig. 5a). We performed automated quantitative image analysis both at single bouton and bouton population levels. These analyses revealed high level of colocalization (Pearson’s coefficient 0.456 ± 0.015; *n* = 52; Fig. 6 optical sections from 3 animals, 2 sections each, sampled evenly from each rat) in the perisomatic region, where inhibitory terminals effectively regulate Na^+^ spike genesis of excitatory neurons (Fig. 6). In contrast, VAMP1 immunolabelling infrequently coincided with VGLUT1 immunostained profiles (Pearson’s coefficient 0.231 ± 0.016, *n* = 58; Fig. 6) in stratum radiatum where excitatory terminals are located (Figs. 5b, 6). VAMP2 expressing synaptic terminals were predominantly VGLUT-1 positive (Pearson’s coefficient 0.542 ± 0.015, *n* = 59) (Figs. 5d, 6), and infrequently found to express GAD65 (Pearson’s coefficient 0.044 ± 0.023, *n* = 60) (Figs. 5c, 6). The neuron specific expression of VAMPs shown by these correlations differed significantly showing that GAD65 correlated significantly more strongly with VAMP1 than VAMP2, and that VLUT1 correlated significantly more strongly with VAMP2 than VAMP1 (*P* < 0.0001 in both cases, unpaired *t* tests, Fig. 6). The highest density of VAMP2 immunopositive axon terminals was in stratum lucidum, which contains the mossy fibre excitatory input onto CA3 pyramidal cells (Figs. 2d, 5d). These results indicate that VAMP1 is expressed preferentially but not exclusively in soma-targeting inhibitory synapses, while VAMP2 is primarily associated with excitatory boutons.

### Functional consequences of tetanus toxin injection on synaptic transmission

**Fig. 7:**
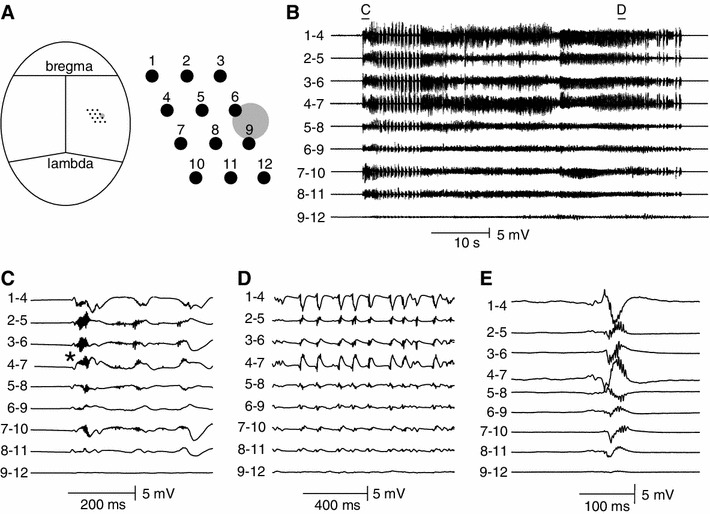
Spatial profile of epileptic activity in freely moving animals. **a** Schematics of tetrode spatial organization in relation to the site of TeNT injection. **b** Recording 13 days after injection shows spontaneous seizures, which involves most of the recording area. A bipolar montage was derived from the reference-mode recording to reveal locally generated activity. Seizures are generated across most of the recording area. **c** Seizure onset is characterised by the presence of slow field potentials with superimposed high-frequency activity at ~280 Hz. Seizure onset site is marked by* star*. **d** Detail of advanced part of seizure. **e** High-amplitude discharges with superimposed high-frequency oscillations (>240 Hz) are present between seizures

The loss of both VAMP1 and VAMP2 suggests that both excitatory and inhibitory neurotransmission would be lost. This appears inconsistent with the evidence in vivo: epilepsy in this model and spastic paralysis with clinical tetanus. It also appears inconsistent with earlier reports on synaptic responses in vitro following intrahippocampal TeNT (Jefferys 1989; Jordan and Jefferys 1992) and with more recent work on spinal neurons (Shin et al. 2012). Given the spatial distribution of changes in VAMP following TeNT injection we first made chronic in vivo recordings from a closely spaced array of metal microelectrodes revealed seizures initiating within 300–450 μm of the injection site (recordings 13 days after injection are shown in Fig. 7). This shows that the VAMP-depleted area around the injection site, spanning ~2.5 mm, was capable of initiating and sustaining seizures, and that this area’s cellular electrophysiology is directly relevant to the epileptic syndrome.

**Fig. 8:**
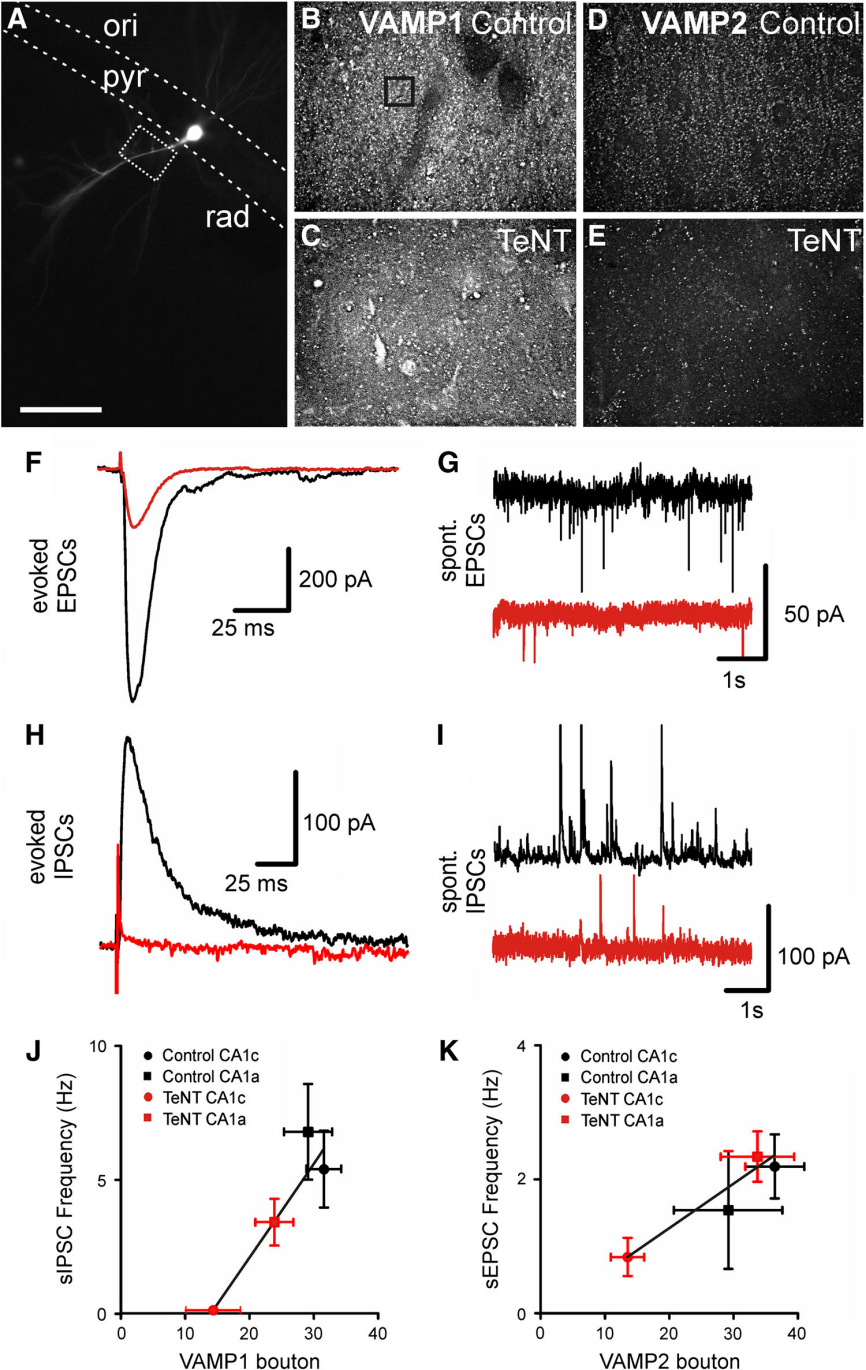
Electrophysiology in CA1c pyramidal cells 8–16 days after injection. **a** Recovered pyramidal cell labelled with biocytin during recording (*scale* 50 μm, *dashed lines* pyramidal layer, *dashed box* area of photomicrographs on **c**/**e**). **b** VAMP1 immunolabelled axon terminals in stratum radiatum adjacent to labelled pyramidal cell in control tissue (slices resectioned for histology; four regions of interest (10 μm^2^, size of *dashed box*) were positioned and boutons were detected and counted). **c** VAMP1 immunoreactive boutons in TeNT-injected rat (as in **b**). **d** VAMP2 immunostained axon terminals in control tissue as in **b**. **e** VAMP2 immunostaining in treated animal, as in **c**. **f**, **g** Evoked and spontaneous EPSCs reveal considerable reductions following injection of TeNT (*red* traces). **h**, **i** Evoked and spontaneous IPSCs reveal near-abolition of following injection of TeNT. **j**, **k** Reductions in frequencies of spontaneous synaptic events correlate strongly with the corresponding VAMP immunopositive bouton count

We prepared hippocampal slices for electrophysiology 8–16 days after intrahippocampal TeNT injection, made whole-cell recordings from pyramidal neurons which were filled with Alexa (Fig. 8a) and counted the number VAMP1 and VAMP2 boutons close to the recorded neurons (Fig. 8b–e) to correlate the morphological and functional measures. In CA1 close to the injection site (CA1c, adjacent to CA3 and inside the area of VAMP cleavage) evoked EPSCs were substantially reduced, to 22 % of control values while IPSCs were abolished (Fig. 8f and h, respectively). Neither measure was affected in CA1a, which is outside the zone of VAMP depletion (Supplemental Figure 2; Supplemental Table 1). To improve the quantification of synaptic function we then recorded spontaneous EPSCs and IPSCs in CA1c. The amplitudes of both types of spontaneous event were not affected by TeNT (Supplemental Table 2), but the frequencies were substantially reduced (Fig. 8g, i). Spontaneous IPSCs fell to very low frequencies, ~2 % of control, while spontaneous EPSCs fell to ~38 % of control (Fig. 8j, k). The relative preservation of EPSCs provides a plausible explanation of the epileptic syndrome, but it raises the question of whether this can be explained by a preservation of VAMP2 in a subset of boutons. We therefore measured the density of VAMP1 and VAMP2 labelled boutons close to the tracer-labelled neurons in the slices used for the electrophysiology. In CA1c, close to the injection, VAMP1 decreased to 50 % of control (*P* = 0.001) and VAMP2 decreased to 53 % (*P* = 0.023). Both evoked and spontaneous PSC frequency positively correlated with the density of VAMP boutons (spontaneous PSCs are shown in Fig. 8j, k; VAMP1-sIPSCs, *r* ^2^ = 0.97, *P* = 0.01; VAMP2-sEPSCs, *r* ^2^ = 0.95, *P* = 0.01; evoked EPSCs are shown in Supplemental Figure 1 and Supplemental Table 1).

In contralateral CA1 no statistical significant deficit was observed in any of the functional measures characterizing excitatory and inhibitory transmission (Supplemental Tables 1, 2). The density of VAMP1 and VAMP2 boutons did not differ between TeNT-injected animals and controls, nor between CA1c and CA1a areas (data not shown). We also determined the bouton density for CA3 bilaterally 8-16 days after injection. TeNT decreased ipsilateral VAMP1 and VAMP2 significantly (respectively to 55 and 65 % of control; *P* < 0.001 and *P* = 0.001). In contrast with the measurements for CA1, in CA3 contralateral to the TeNT injection the median VAMP1 bouton density decreased significantly to 76 % of control (*P* = 0.009, Mann–Whitney rank sum) and the median VAMP2 decreased to 65 % of control (*P* = 0.001, Mann–Whitney rank sum).

### Increased VAMP1 expression in inhibitory cell somata

**Fig. 9:**
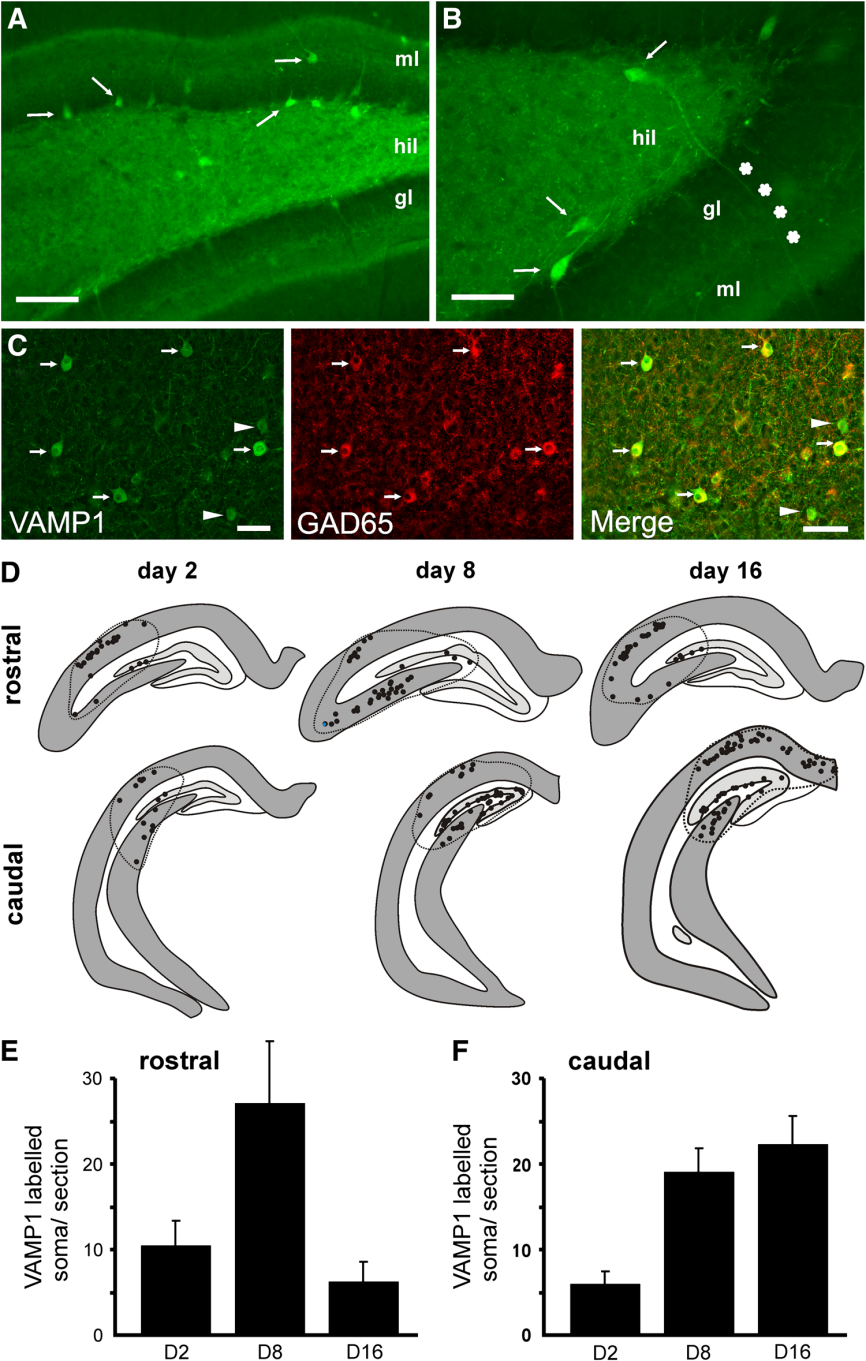
VAMP1 overexpression and accumulation in inhibitory cell somata after TeNT injection. **a**, **b** VAMP1 immunolabelling identifies cells with high levels of VAMP1 expression in the somata in the dentate gyrus of the TeNT-injected hippocampus (*arrows*). **c** Double immunohistochemical labelling shows high degree of colocalization of VAMP1 and GAD65. *Arrows* indicate colocalization, arrowheads occasional lack of colocalization between the two signals. **d** Spatiotemporal distribution of the VAMP1 immunopositive cell bodies. They appeared frequently around the area of VAMP1 depletion (*dashed lines*). **e**, **f** Spatiotemporal distribution of the numbers of VAMP1 overexpressing cells in rostral (**e**) and caudal parts of the dorsal hippocampus (**f**). *Scale bars* **a** 100 μm, **b**, **c** 50 μm

We observed VAMP1 immunopositive somata within the region of depletion of synaptic VAMP1 in the vicinity of the TeNT injection (Fig. 9). A total of 1,807 VAMP1 expressing cell bodies were identified from 120 hippocampal sections prepared from all three survival times following TeNT injection. No VAMP1 immunopositive somata were present in control tissue or in regions remote from the injection site in rats treated with TeNT. These VAMP1-positive somata appeared in all hippocampal subregions as long as they were within the area of toxin-induced loss of VAMP (Fig. 9d). In some cases major dendrites were also immunopositive for VAMP1 (Fig. 9a–c). The greatest number of labelled cells (27.0 ± 7.4) was observed in rostral sections of the day 8 group (Fig. 9e). In the caudal hippocampus the largest number of labelled somata (22.5 ± 3.4) was reached 16 days after toxin injection (Fig. 8f). VAMP1 expressing cell bodies were found principally in the stratum pyramidale, at the border of the strata radiatum and pyramidale and occasionally in strata oriens and lacunosum moleculare in CA1 and CA3 (Fig. 9d). In dentate gyrus they were located at the molecular layer and at the border of the granule cell layer and in the hilus (Fig. 9a, b).

The majority of VAMP1 positive cells were characterised by round, occasionally elongated cell bodies (Fig. 9a–c), and dendrites were aspiny with a beaded appearance especially at their thinner distal regions. Based on their morphological features 98 % of the analysed cells could be classified as inhibitory neurons, including likely basket cells which were common in strata pyramidale and radiatum. This was confirmed by colocalization of GAD65 in 87 % of the VAMP1 positive cell bodies (only 6 % showed no clear colocalization with GAD65).

**Fig. 10:**
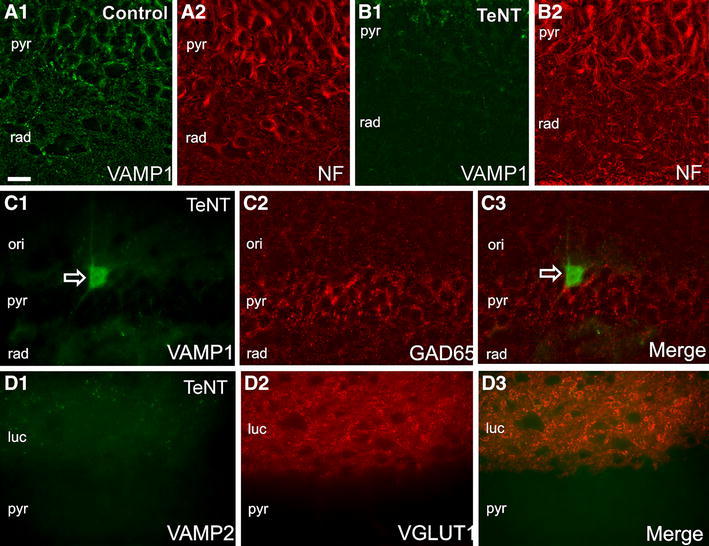
TeNT cleaves VAMPs but neither neurofilament nor enzymes related to production and packaging of neurotransmitters. **a** Double immunofluorescent labelling of Neurofilament 68 k (NF, **a1**) and VAMP1 (**a2**) in control hippocampus. **b** TeNT injection causes loss of VAMP1 immunosignal (**b1**) but does not affect NF expression (**b2**). **c** Double immunofluorescent labelling illustrates selective cleavage of the VAMP1 (**c1**) while GAD65 expression is unaffected (**c2**, **c3**). Note VAMP1 immunolabelled soma (*open arrow* **c1**, **c3**) despite lack of VAMP1 positive axon terminals. **d1**–**d3** TeNT injection also depletes VAMP2 without loss of VGLUT1 expression; somata are not labelled with VAMP2. *ori* stratum oriens, *pyr* stratum pyramidale, *luc* stratum lucidum, *rad* stratum radiatum. *Scale bar* 20 μm

We tested whether VAMP1 accumulation in somata and dendrites resulted from damage to axoplasmic transport by double immunolabelling for VAMP1 and neurofilament 68k, the anatomical substrate of the intra axonal transport system (Fig. 10a, b). Immunolabelling showed loss of VAMP1 but no change in neurofilament immunolabelling, even in the centre of the VAMP depletion zone close to the injection site. Thus, accumulation of VAMP1 in inhibitory cell somata is most likely the result of enhanced protein synthesis rather than damage of transport.

### Survival of synaptic terminals after VAMP cleavage by tetanus toxin

Depletion of both isoforms of VAMP occurs close to the site of TeNT injection by day 2, although only in the case of VAMP1 is this extensive enough to be detected by the grey scale analysis. Immunolabelling of this focal region of cleavage reveals near-complete loss of both VAMP1 and VAMP2 (Fig. 10c, d). However, in both cases VGLUT and GAD65 expression is preserved, suggesting that the presynaptic terminals of both excitatory and inhibitory neurons survive loss of VAMPs.

## Discussion

Our immunohistochemical observation of the distribution of VAMP isoforms in the hippocampus extends previous reports on expression in the brain as a whole (Raptis et al. 2005). The spatial separation of inhibitory and excitatory inputs in the hippocampus allowed us to determine the coexpression of each VAMP isoform with specific markers for excitatory and inhibitory synapses, quantifying the correlations of the pairs of proteins (Bolte and Cordelieres 2006). We analysed the VAMP expression in inhibitory terminals in the perisomatic region where the axon terminals are almost exclusively inhibitory and predominantly from basket cells, while the VAMP expression of excitatory boutons were analysed in stratum radiatum, adjacent to stratum pyramidale, where the excitatory Schaffer collaterals dominate the input. We found much more prevalent expression of VAMP1 than VAMP2 in hippocampal pyramidal layer inhibitory synapses (Fig. 6) contrasting with the near-identical colocalization of the two VAMP isoforms in neocortical inhibitory synapses (Bragina et al. 2010). VAMP2 was predominantly expressed in excitatory synapses in the hippocampus, similar to the neocortex (Bragina et al. 2010). The difference between hippocampal and neocortical inhibitory synapses could be due to: the more specific sampling of soma-targeting synapses in the hippocampus, or to neocortical and hippocampal synapses having different biochemical machinery for GABA release (Sugino et al. 2006), or to the different inhibitory cell markers used in the two studies, respectively GAD and VGAT (Bragina et al. 2010). A lack of VGAT expression has been reported for some GABA-expressing neurons (Chaudhry et al. 1998; Zhao et al. 2011; Jarvie and Hentges 2012).

In the present study intrahippocampal injection of TeNT caused substantial focal reductions in both VAMP1 and VAMP2, providing evidence that rat VAMP1 can indeed be a substrate for TeNT in rat brain, in contrast with previous reports (Schiavo et al. 1993) which continue to inform work on the topic (Chen et al. 2012; Mainardi et al. 2012). The depletion by TeNT of both VAMP1 and VAMP2 to similar degrees argues against another hypothesis for the clinical effects of the toxin, that greater uptake into the more rapidly firing inhibitory neurons can explain a selective effect of TeNT on inhibitory transmission (Williamson et al. 1992), however, this conclusion must be qualified by the unreliability of comparing two different proteins by the binding of two different antibodies. VAMP1 is depleted close to the injection track a few days before VAMP2 (compare Figs. 3 and 4), but epileptic seizures start, within the zone of depleted VAMP, after day 8 when both isoforms have decreased. TeNT uptake does depend on synaptic activity exposing the SV2, to which the toxin binds, to the extracellular space (Yeh et al. 2010), so neurons need to be active, which they are in vivo. Given that some classes of interneuron fire at much higher frequencies than pyramidal cells the results of Williamson et al. (1992) and Yeh et al. (2010) would be consistent with a greater effect of TeNT on inhibition. Curiously, a recent report on acute applications of TeNT to neocortical neurons in culture described a more rapid loss of miniature EPSCs than IPSCs (Yeh et al. 2010). Heterogeneity in expression of major protein families (Sugino et al. 2006) might explain the difference between our study and Yeh’s report (2010).

The model has a sequence of stages, as shown by previous physiological studies (Whittington and Jefferys 1994). Postsynaptic receptor changes have not been extensively investigated, but we do know that responses to exogenous GABA do not change at any stage of the model (Whittington and Jefferys 1994) and that GluR2 receptors show a transient increase in their flip isoform 4 weeks after TeNT injection (Rosa et al. 1999), later than the period covered by the present study. Intrinsic properties of neurons have not been extensively reported in this model: at several months there is evidence of decreased excitability, probably related to decreased densities of Na^+^ channels (Brace et al. 1985; Vreugdenhil et al. 2002), but action potentials look qualitatively normal during the active seizure phase both in pyramidal cells (Jefferys 1989) and interneurons (G.L. Morris, A.D. Powell, P. Jiruska and J.G.R. Jefferys, unpublished). The initial stage of the direct action of the toxin may induce adaptive changes, including increased production that could produce the VAMP1 accumulation in somata reported here. This could be related to the increase in GAD mRNA found around 4 weeks after TeNT injection (Najlerahim et al. 1992), with both changes contributing to the restoration of transmitter release from inhibitory synapses found at later stages in the model (Whittington and Jefferys 1994).

In the present study, we have explicit evidence of a selective disruption of inhibition and relative sparing of excitation within the tissue exposed to the effects of TeNT. Between days 8 and 16 we found that inhibitory synaptic transmission close to the injection site was reduced to ~1 % of control, while excitatory synaptic activity was present, but reduced to 23 % of control. Neurons outside the zone of VAMP depletion retained normal inhibitory and excitatory synaptic activity.

The relative selectivity of TeNT for inhibitory, and relative sparing of excitatory, transmission would explain the clinical and experimental consequences of TeNT in the CNS. However, the loss of VAMP2 immunolabelling quantified by numbers of positive boutons is about the same as the loss of VAMP1 positive. In both cases the frequency of spontaneous PSCs correlates with the density of the corresponding VAMP boutons, but the relationship is steeper for sIPSCs and VAMP1, and importantly approaches zero IPSCs with exposure to TeNT. The reasons for the differences in relationships between synaptic currents and the respective VAMP isoforms are unclear: comparing two proteins by immunohistochemistry is unsafe because the binding characteristics may differ considerably, but different classes of neuron can differ in their release machinery (Song et al. 2013) and it is possible that sensitivities to VAMP depletion may also differ.

The present study questions previous work predicting that rat VAMP1 should not be sensitive to TeNT (Schiavo et al. 1992, 1993). It demonstrates that VAMP1 is mainly in inhibitory boutons and VAMP2 is mainly in excitatory. The relative sparing of spontaneous EPSCs is surprising given the similar degrees of depletion of both VAMP1 and VAMP2, but is related to a steeper relationship following exposure to TeNT of spontaneous IPSCs with density of VAMP1 positive terminals than of EPSCs with density of VAMP2 terminals. This relative sparing of EPSCs provides a basis for the initial stages of the TeNT model of focal epilepsy and, if replicated in motor structures, may help to explain the spastic paralysis characteristic of tetanus intoxication.

## Electronic supplementary material

Below is the link to the electronic supplementary material.

Supplementary material 1 (PDF 423 kb)
