# Supplementary material for: Structural and functional substrates of tetanus toxin in an animal model of temporal lobe epilepsy
Source: Brain Struct Funct. 2014 Jan 18;220(2):1013–29. doi: 10.1007/s00429-013-0697-1 (PMC4341026; doi:10.1007/s00429-013-0697-1)
Supplement: Supplementary file 1 — Supplementary material 1 (PDF 423 kb) [file 429_2013_697_MOESM1_ESM.pdf]

Brain Structure and Function

Structural and functional substrates of tetanus toxin in an animal model of temporal lobe epilepsy

Authors: Alex S. FERECSKÓ[1†*], Premysl JIRUSKA[1,2,3*], Lucy FOSS[1], Andrew D. POWELL[1], Wei-Chih CHANG[1], Attila SIK[1#] and John G.R. JEFFERYS[1#]

Authors affiliations:

[1] Neuronal Networks Group, School of Clinical and Experimental Medicine, University of Birmingham, Birmingham B15 2TT, United Kingdom

[2] Department of Developmental Epileptology, Institute of Physiology, Academy of Sciences of Czech Republic, Prague, CZ-14220, Czech Republic

[3]Department of Neurology, Charles University, 2nd School of Medicine, University Hospital Motol Prague, CZ-15006, Czech Republic

Correspondence to:-

j.g.r.jefferys@bham.ac.uk

a.sik@bham.ac.uk

Supplemental Figure 1: grey scale methods

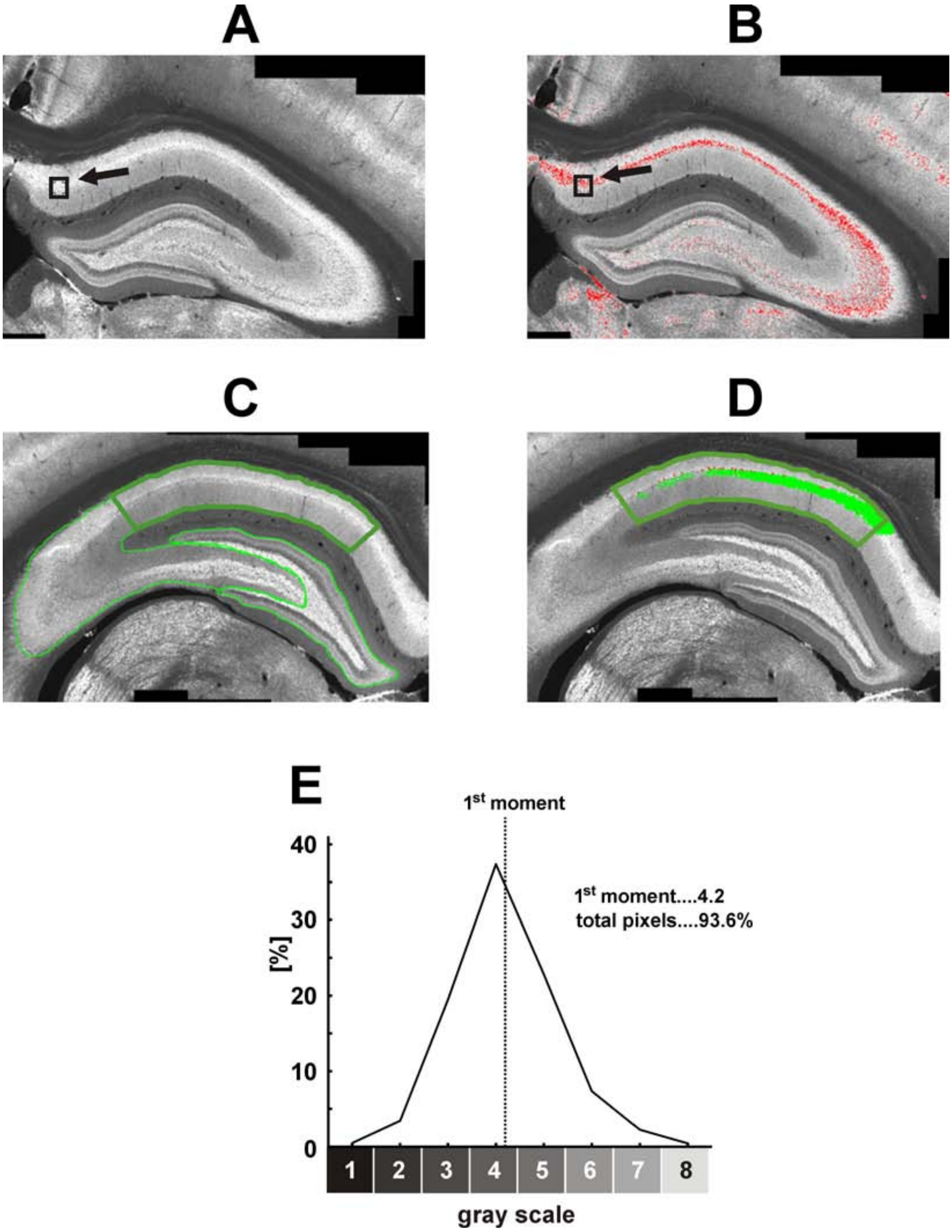

Supplemental figure 1. Quantification of VAMP lesion. A: VAMP immunolabelling in each slice was normalized by the labelling intensity in contralateral subiculum. B: Pixels of specific intensity range are marked red. C: Margins (borders) of three regions of interest: CA1, CA3 and dentate gyrus. D: Number of pixels in each intensity range was converted to area. E: Normalized immunolabelling was distributed into eight bins of grey scale intensity. X-axis represents eight bins of grey colour spectrum. Y-axis represents percentage of pixels distributed in each bin of grey spectrum from the total number of pixels in the studied area. In this example the highest number of pixels was found in bin No. 4. The first moment (m1) of the intensity spectrum was 4.2.

Supplemental Figure 2: synaptic physiology and VAMP+ punctae

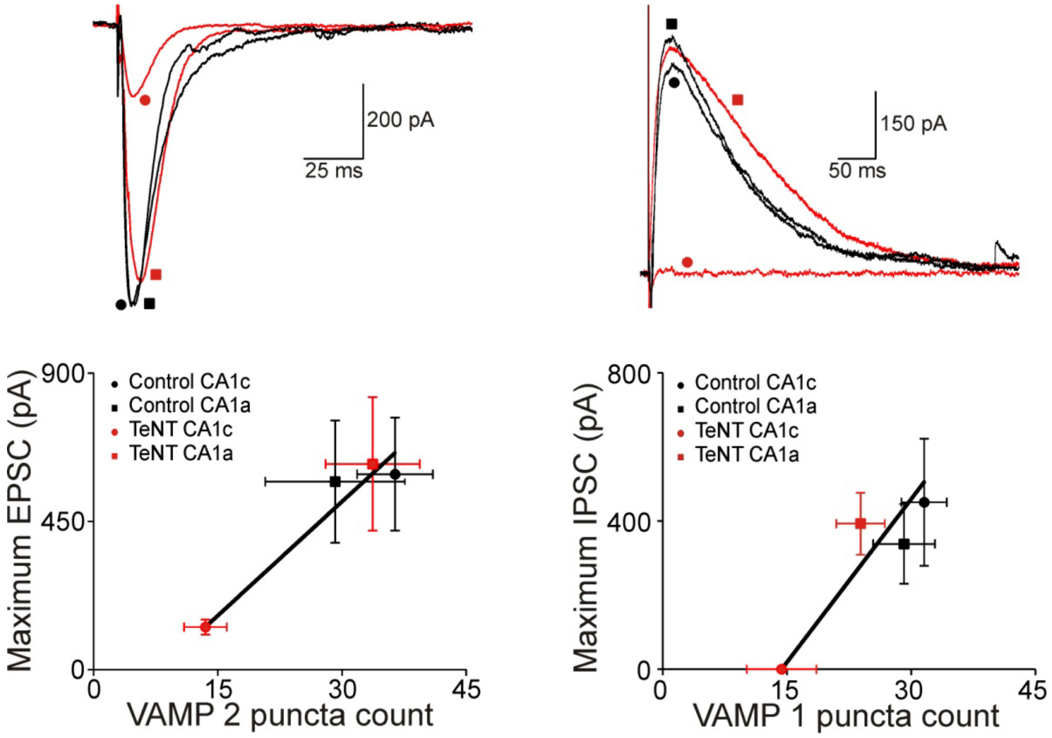

Supplemental figure 2. Whole cell recordings were made from CA1 pyramidal neurons in hippocampal slices prepared 8-16 days after intrahippocampal injection of tetanus neurotoxin (TeNT) or control (vehicle) solution as described in the Methods. Synaptic currents were evoked by stimulation of stratum radiatum. Injection of TeNT considerably decreased evoked EPSCs in area CA1c, close to the injection site (Fig 7F; Supplemental Fig 2, top left). This toxin-dependent decrease did not apply to pyramidal cells recorded in CA1a, which were outside the limits of the zone of VAMP depletion (Supplemental Fig1, left panels; Supplemental Table 1). Equivalent recordings of IPSCs revealed that TeNT effectively abolished the responses from CA1c (Fig 7H; Supplemental Fig 2 top right) but not from CA1a (supplemental Fig 2, right panels) nor from CA1 contralateral to the injection site (Supplemental Table 1).

Supplemental Table 1. Evoked Synaptic currents

	Ipsilateral			
	Control		Epileptic	
	CA1a (n=7)	CA1c (n=8)	CA1a(n=5)	CA1c(n=5)
**max EPSC amplitude (pA)**	570.5±185.6	593.6±171.4	624.6±202.5	**129.1±22.7(*)**
**V50 (V)**	52.8±8.9	48.9±3.8	52.7±2.7	57.1±9.4
**Slope (pA/V)**	0.05±0.01	0.05±0.01	0.04±0.004	0.09±0.01

	Contralateral			
	Control		Epileptic	
	CA1a (n=6)	CA1c (n=5)	CA1a(n=4)	CA1c(n=5)
**max EPSC amplitud (pA)**	201.2±63.0	263.7±42.8	310.1±29.4	168.1±57.6
**V50 (V)**	49.1±7.1	49.1±3.0	51.6±0.6	60.4±12.7
**Slope (pA/V)**	0.07±0.01	0.05±0.01	0.05±0.01	0.05±0.01

	Ipsilateral			
	Control		Epileptic	
	CA1a (n=7)	CA1c (n=8)	CA1a(n=5)	CA1c(n=5)
**max IPSC amplitude (pA)**	338.3±107.6	392.8±83.7	450.5±171.4	0±0(***)
**V50 (V)**	28.4±11.1	38.0±8.6	30.6±3.6	0±0(***)
**Slope (pA/V)**	0.07±0.004	0.06±0.03	0.07±0.02	0±0(***)

	Contralateral			
	Control		Epileptic	
	CA1a (n=6)	CA1c (n=5)	CA1a(n=4)	CA1c(n=5)
**max IPSC amplitude (pA)**	158.7±35.1	207.2±76.9	256.8±89.1	171.2±45.0
**V50 (V)**	43.0±10.7	46.5±5.8	47.1±9.4	42.4±3.9
**Slope (pA/V)**	0.06±0.007	0.03±0.003	0.04±0.009	0.07±0.03

Supplemental Table 2. Spontaneous synaptic currents

	Ipsilateral			
	Control		Epileptic	
	CA1a (n=5)	CA1c (n=7)	CA1a(n=11)	CA1c(n=7)
**Spontaneous EPSC amplitude (pA)**	23.4±0.3	23.0±3.4	25.8±2.7	26.0±3.4
**Spontaneous IPSC amplitude (pA)**	20.0±0.6	25.4±2.7	20.5±1.8	24.2±3.8

	Contralateral			
	Control		Epileptic	
	CA1a (n=5)	CA1c (n=9)	CA1a(n=9)	CA1c(n=6)
**Spontaneous EPSC amplitude (pA)**	12.7±0.5	16.9±1.8	19.2±5.1	14.3±2.4
**Spontaneous IPSC amplitude (pA)**	13.7±0.1	16.3±1.7	18.3±2.4	16.2±2.4

Recordings of spontaneous synaptic currents revealed substantial decreases in frequencies of both IPSCs (Fig 7H, I, J) and EPSCs (Fig 7F, G, K). The amplitudes of spontaneous EPSCs and IPSCs were not affected by injection of TeNT in CA1a or CA1c in either ipsilateral or contralateral (uninjected) hippocampus.
